# Application of radiomics in lung immuno‐oncology

**DOI:** 10.1002/pro6.1191

**Published:** 2023-04-04

**Authors:** Weisi Yan, Chen Quan, Waleed F. Mourad, Jianda Yuan, Zheng Shi, Jun Yang, Qiuxia Lu, Jie Zhang

**Affiliations:** ^1^ Baptist Health System Lexington Kentucky USA; ^2^ City of Hope Comprehensive Cancer Center Duarte California USA; ^3^ Department of Radiation Medicine University of Kentucky Lexington Kentucky USA; ^4^ Translational Oncology at Merck & Co Kenilworth New Jersey USA; ^5^ UMC Health System Lubbock Texas USA; ^6^ Foshan Chancheng Hospital Foshan Guangdong China; ^7^ Department of Radiology University of Kentucky Lexington Kentucky USA

**Keywords:** immune modulation, non‐small cell lung cancer, radiation, radiomics

## Abstract

Radiomics is a rapidly evolving field of research that extracts and analyzes quantitative features within medical images. Those features are termed as radiomic features that can characterize a tumor in a comprehensive and quantitative manner with regard to its internal structure and heterogeneity. Radiomic features can be used, alone or in combination with demographic, histological, genomic, or proteomic data, for predicting prognosis or treatment response. Immunotherapy, or immune‐oncology, is the study of cancer treatment by taking advantage of the body's immune system to prevent, control, and eliminate cancer. In this review, we first provide a brief introduction to both radiomics and immune‐oncology in lung cancer. Then, we discuss the need for developing immune‐oncology biomarkers, and the advantages of radiomics in identifying biomarkers related to immunotherapy. We also discuss potential areas in and out of tumors, such as the intra‐tumoral hypoxic region and tumor microenvironment, where radiomic markers might be extracted, as well as a potential application of radiomic biomarkers in clinical lung cancer management. Finally, we present radiation and immune modulation in non‐small cell lung cancer, clinical trials and their design to incorporate radiomic biomarkers, and radiomics‐guided precision radiation therapy.

## Graphical abstract

Radiomics is a rapidly evolving field of research that extracts and analyzes quantitative features within medical images. Those features are termed as radiomic features that can characterize a tumor in a comprehensive and quantitative manner with regard to its internal structure and heterogeneity. Radiomic features can be used, alone or in combination with demographic, histological, genomic, or proteomic data, for predicting prognosis or treatment response. Immunotherapy, or immune‐oncology, is the study of cancer treatment by taking advantage of the body's immune system to prevent, control, and eliminate cancer. In this review, we first provide a brief introduction to both radiomics and immune‐oncology in lung cancer. Then, we discuss the need in developing immune‐oncology biomarkers, and the advantages of radiomics in identifying biomarkers related to immunotherapy. We also discuss potential areas in and out of tumors, such as intra‐tumoral hypoxic region and tumor microenvironment, where radiomic markers might be extracted, as well as potential application of radiomic biomarkers in clinical lung cancer management. Finally, we present radiation and immune modulation in non‐small cell lung cancer, clinical trials, and their design to incorporate radiomic biomarkers, and radiomics‐guided precision radiation therapy.

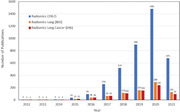

## 1 RADIOMICS: THE BASICS

**FIGURE 1:**
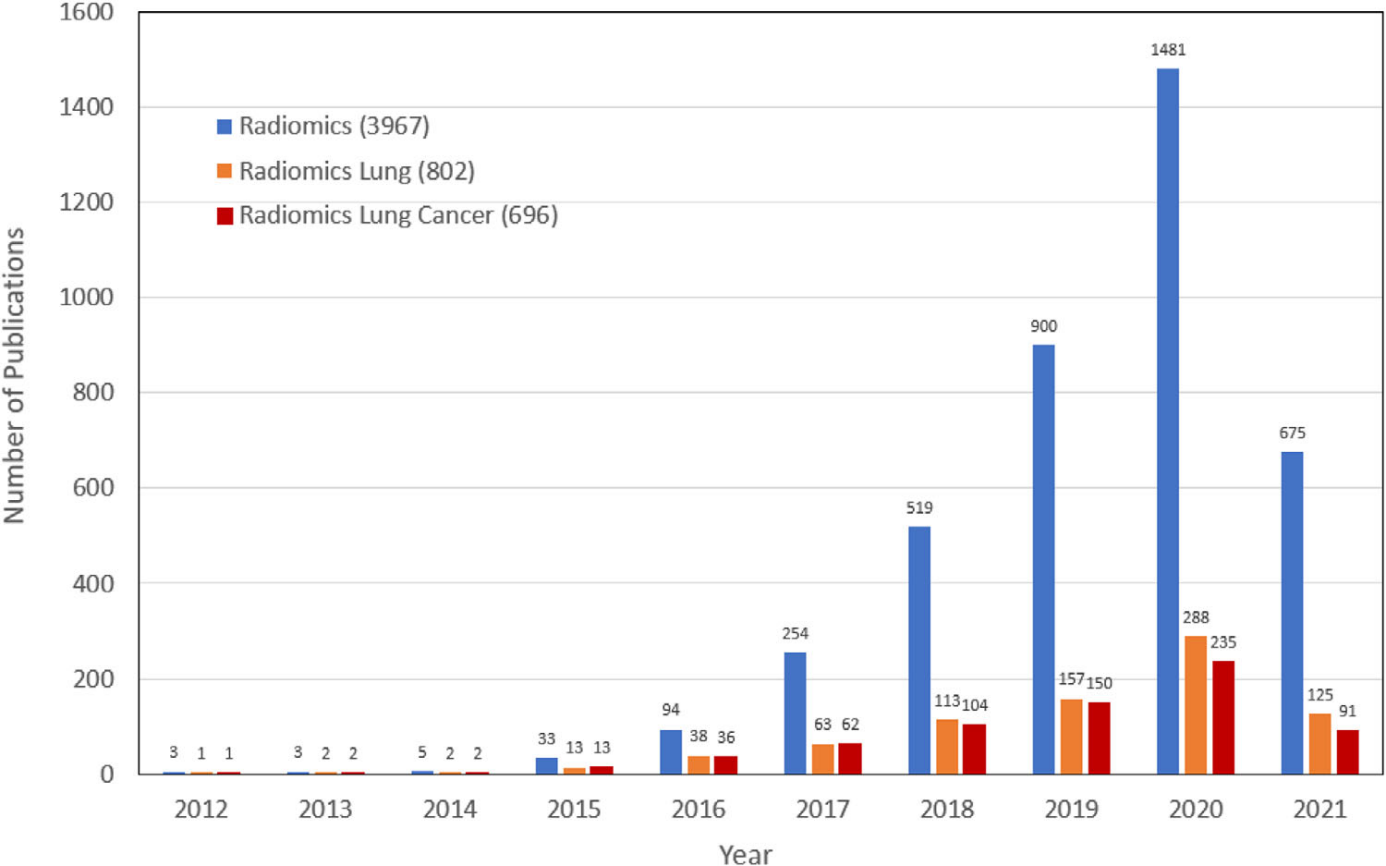
The number of publications on radiomics and radiomics lung extracted from PubMed database (accessed on April 15, 2021).

Radiomics uses quantitative methods to extract and analyze features from existing medical images or radiological databases. The concept of radiomics was first proposed in 2012.^1^ Since then, radiomics study has experienced exponential growth. We have searched the publications for radiomics in oncology from the PubMed database and collected corresponding data, with approximately 20% of the studies focusing on the lung (Figure 1).

Radiomics can be used to characterize a tumor in a comprehensive and quantitative manner by means of radiological image analysis with regard to its internal structure and heterogeneity. This overcomes the limit of biopsy in that the biopsy tissue can frequently only be obtained from a portion of tumor, thus being not representative, or even obtained from non‐lesional areas. Thus, radiomics could potentially provide a more comprehensive analysis of the tumor phenotype to create a virtual reconstruction/analysis of a tumor in a holistic, whole, and functional manner (there were 2819 publications in the year of 2022).

Radiomic features are able to serve as a tool for personalized diagnosis, treatment guidance, and response monitoring in clinical practice. Studies of radiomics have been used in head and neck, and lung cancers to identify prognostic phenotypes,^2^ predict clinical outcomes in early‐stage non‐small cell lung cancer (NSCLC) patients treated with stereotactic body radiation therapy,^3^ and assist in immunotherapy by estimating CD8 cell infiltration into the tumor and predicting clinical outcomes.^4^, ^5^

Studies using radiomics in lung cancer have generated radiomic signatures with markers to predict metastasis and survival.^5^ The analysis of tumor heterogeneity has suggested that tumor biology can be reflected through tumor shape and intratumor density variation, from which imaging characteristics can be used to predict tumor development and patient survival.^6^

## 2 IMMUNO‐ONCOLOGY IN LUNG CANCER

Improved understanding of cancer has resulted in the development and clinical immunology checkpoint blockade antibodies targeting the cytotoxic T‐lymphocyte‐associated protein 4, programmed death‐1 (PD‐1), and programmed death‐ligand (PD‐L1) axis. Through modulating T‐cell activation and exhaustion, these antibodies have shown impressive disease control and promising responses in patients with multiple solid tumor types.^7^, ^8^

Immune checkpoint inhibitors (ICIs) work on the patient's own immune system to enhance the actions of tumor‐specific cytotoxic T cells. An independent prognostic marker of survival for patients with NSCLC is the presence of tumor‐infiltrating lymphocytes in cancer cells.^9^ Pembrolizumab, a monoclonal immunoglobulin antibody against PD‐1, was recently approved by the US Food and Drug Administration for monotherapy for patients with stage III NSCLC who are not candidates for surgical resection or definitive chemoradiation, or have metastatic NSCLC with no epidermal growth factor receptor (EGFR) or anaplastic lymphoma kinase genomic tumor aberrations. This is based on findings from the KEYNOTE‐042 trial.^10^ Subsequently, the combination of chemotherapy with checkpoint inhibitor was tested. KEYNOTE 189 and 047 reported that the addition of pembrolizumab to chemotherapy resulted in a clear overall survival (OS) and progression‐free survival benefit. OS benefit was observed across all patient subgroups, including a cohort without PD‐L1 expression.^11^, ^12^

Currently, there is a clinical need for identifying biomarkers that predict response and help selection for an appropriate combination of therapies.^13^, ^14^

### 2.1 Need for developing immuno‐oncology biomarkers

So far, only PD‐L1 IHC has been approved as a diagnostic biomarker by the US Food and Drug Administration for patients with advanced NSCLC for PD‐L1 status and patient selection for PD‐1/PD‐L1‐directed therapy.^15^ However, PD‐L1 seems to be not the only biomarker to predict response. There are still many uncertainties about the practical value of PD‐L1 in the clinical practice of ICI in untreated NSCLC. For example, pembrolizumab is known to be effective in patients with PD‐L1 expression >50%. Interestingly, there is a subgroup of patients who benefited from pembrolizumab with chemotherapy with PD‐L1 expression <1%.^12^ Identifying biomarkers in this subset of patients with low PD‐L1 expression, but who could benefit from pembrolizumab, would be of interest.

For patients with higher PD‐L1 levels (expression range of 50%–100%), the overall response rate was 44.4%, indicating >50% of patients with high PD‐L1 levels would not respond to immune checkpoint inhibition as expected. Responses and clinical outcomes were improved with PD‐L1 expression levels ≥90%.^16^ Therefore, additional biomarkers for patients with PD‐L1 expression levels <90% would be desirable. Furthermore, mechanisms exist beyond tumor PD‐L1 staining from a biopsy that can monitor/predict possible immune responses. Recent data have shown that tumor‐infiltrating immune cell PD‐L1 expression has a stronger association with treatment response than that of tumor cell PD‐L1 expression.^17^ The baseline density and location of CD8^+^ T cells at the margin or the core of tumor, and the pre‐existing CD8 T cells that are negatively regulated by PD‐1/PD‐L1 could determine antitumor activity by PD‐1 immune checkpoints.^18^

Regulatory agencies and professional societies, such as the Society for Immunotherapy of Cancer, have recognized the need for developing immuno‐oncology biomarkers for clinical use.^19^ Immune biomarkers will monitor disease progression, decide when to alter/stop costly immunotherapy treatment, and potentially enable a better selection of patients for cancer immunotherapies while avoiding adverse events.

### 2.2 Advantages of radiomics in identifying immunotherapy‐relevant biomarkers

Several tumor‐derived biomarkers have been reported, such as PD‐L1 expression on tumor cells (and immune cells), tumor mutational load/burden, DNA mismatch repair genes and their products, and multigene signatures (tumor microenvironment).^20^ These tumor‐based biomarkers are inherently subject to sampling bias. Next‐generation sequencing and bioinformatics have shown that there is heterogeneity intratumorally, which suggests that the tumor is not homogenous, but a complex system.^21^ Multiple site biopsies have revealed that there is spatial composition and different evolution of different subclones in a given tumor.^22^, ^23^, ^24^, ^25^ Hence, a single biopsy or acquiring material for biomarker analysis will lead to omitting intratumor heterogeneity and other subclones, which generate either limited information or lead to rapid resistance when resistance mutations were not detected in the sampling process.^26^, ^27^

Alternatively, radiomics could complement these biomarkers and, thus, lead to a more precise, multimodal prediction. As aforementioned, the study of spatial intratumor heterogeneity is an inherent nature of radiomics, which can see the whole tumor in a whole elephant approach and avoid the sample limitation from a simple tumor biopsy or limited sampling from the whole tumor. Radiomics cannot detect the biological ancestry of cells in a given tumor yet, but can detect areas that harbor different clonal populations intratumorally. It also has the potential to be combined with liquid biopsy to extract valuable information in determining tumor aggressiveness.^28^

Application of radiomics in evaluating tumor immune cell infiltration and predicting response to anti‐PD‐1 therapy has been promising: a radiomic signature that measures the intensity of tumor‐infiltrating CD8 cells was identified. This CD8 cell signature was validated with pathologists quantifying tumor‐infiltrating CD8 lymphocytes from tissue slides from biopsy samples corresponding to the primary tumor and matching computed tomography (CT) scans from The Cancer Genome Atlas. After confirmation of validity, this signature was used to evaluate patients with advanced solid tumors treated in phase I trials of PD‐1/PD‐L1 monotherapy, which showed that a higher score in this signature is related to longer OS.^4^

Recently, Gilles et al. used the multiparametric radiomics signature from features extracted from positron emission tomography (PET)/CT images to predict the response to immunotherapy. Their findings suggested that tumors that contain radiomics signatures associated with more heterogeneous tumors, more convexity and lower mean standard uptake value and Hounsfield unit had a larger probability of responding to immunotherapy. Also, the predictive result with an area under the curve of 0.82 showed promise of the radiomics signature being used as a predictive biomarker to guide immunotherapy.^29^

### 2.3 Potential application of radiomic biomarkers in lung cancer management

Radiomics‐identified biomarkers can potentially be used to guide treatment decisions, predict prognosis, and/or monitor treatment response. With prognostic and predictive immune biomarkers, clinicians can assess the responses throughout treatment, predict patient benefits from therapeutic agents, monitor their response, and enable personalized treatment plans.

For example, the association of CT features and genetic markers, such as the mutational status of the *KRAS* mutation and *EGFR* mutation in NSCLC, has been studied.^30^ There are some potential features (shape, pleural retraction, internal air bronchogram) that could suggest a correlation with genetic changes. Several other groups conducted CT‐based studies to predict the *EGFR* mutation in lung adenocarcinoma.^31^, ^32^, ^33^ One recent prediction model was built on radiomics features, such as shape and size, and textual and wavelet features. The AUC value was 0.802. However, the sensitivity, specificity, and accuracy were still not satisfactory for clinical use.^33^

Radiomics is non‐invasive, and can be performed readily in a rapid manner to predict or monitor treatment response. Radiomics analysis has shown that abnormal texture presented on baseline 18F‐fluorodeoxyglucose PET, such as coarseness, contrast, and busyness, are associated with non‐response to chemoradiotherapy and with poorer prognosis. A prognostic radiomic signature that correlated to intratumor heterogeneity is associated with gene‐expression patterns and proliferation of tumors.

### 2.4 Potential areas in and out of tumor that may provide radiomic biomarkers

#### 2.4.1 Intratumor hypoxic regions

Hypoxia is a common scenario among solid tumors. Indeed, it enables many events in the tumor microenvironment that lead to the expansion of aggressive clones that adapt to hypoxia. Hypoxia in tumors is related to poor clinical prognosis,^34^, ^35^ elevated genomic instability,^36^ resistance to radiation therapy,^37^, ^38^ chemotherapy^38^ and immunotherapy,^39^, ^40^ and even a poor outcome.^41^

Hypoxia is known to contribute to the upregulation of the expression of PD‐L1 and establish an immunosuppressive tumor microenvironment.^42^ Also, hypoxia harms CD8^+^ T cells *in vitro* and global antitumor immune response *in vivo*.^40^ The efficacy of the PD‐1 blockage is potentiated by the metformin‐induced reduction of tumor hypoxia in preclinical mice models.^39^ Furthermore, metformin also enhances the PD‐1 blockade through a reduction of tumor hypoxia. Patient outcomes are improved with metformin in combination with ICIs without reaching significance, which hints that reversing hypoxia can contribute to better immunotherapy outcome.^43^ Radiation studies that targeted hypoxic regions of tumors contribute to improved tumor abscopal response without the use of ICIs.

Fluoromisonidazole PET can detect tumor hypoxia, but it is not widely available.^44^ Radiomics methods have been shown to be able to identify hypoxic regions in tumors. It has been shown that imaging features extracted from CT and 18F‐fluorodeoxyglucose PET imaging can be correlated with the magnitude of hypoxia in tumors as detected by fluoromisonidazole PET.^45^ The collection of radiomics signatures from these hypoxic regions might provide biomarkers for immune resistance and be used to select tumors that are not likely to respond to immunotherapy without the need of specialized imaging devices, and can be applied to general CT images.

#### 2.4.2 Tumor microenvironment

The microenvironment surrounding the tumor mass is a dynamic field of tumor growth, and ongoing interaction of malignant cell expansion and cells that work against this progress.^46^ A study of tumor the margin in clinical human colorectal cancers has yielded interesting findings that a high density of immune cells infiltration in both the center of the tumor and invasive margin is correlated with favorable prognosis, regardless of the local extent of the tumor of metastatic disease in the lymph nodes. The type, density, and location of immune cells in colorectal cancer have a better prognostic value independent of the current tumor, node, and metastasis classification.^47^

Recently, studies have been directed to tumor microenvironments (TMEs). Larger numbers of tumor‐associated inflammatory cells were observed in the peritumoral compartment than in the intratumoral region, according to NSCLC histological specimens, and more tumor‐associated inflammatory cells were associated with favorable disease outcomes, such as improved recurrence‐free survival.^48^ Peritumoral radiomic features extracted from baseline magnetic resonance imaging studies were suggested to be predictive for pathological complete response to neoadjuvant chemotherapy in patients with breast cancer.^49^ In addition, another study of peritumor texture patterns generated signatures that can predict the distant metastatic potential of tumors.^50^

Thus, studying radiomics signatures related to CD8 T‐cell infiltration in these areas would provide hints for the strength of immune response. While studying CD4^+^ regulatory T cells signatures might yield information about tumor immune evasion.^51^

## 3 RADIOMICS AND IMMUNO‐ONCOLOGY

### 3.1 Radiation and immune modulation in NSCLC

Radiation therapy is an integral part of lung cancer management. Stereotactic body radiation therapy is used for early‐stage lung cancer,^52^ whereas chemoradiation is reserved for locally advanced NSCLC.^53^ The application of radiation therapy is fairly limited in stage IV lung cancers, mainly for palliation purposes. Approximately 40% of patients with NSCLC are diagnosed with stage IV lung cancer on diagnosis.^54^ These patients are frequently treated with PD‐1 checkpoint inhibitors,^55^ and radiation has been suggested to play a synergistic effect with anti‐PD‐1 therapy.^56^ Thus, radiation doses have the potential to be better utilized as a trigger for immune priming. Radiomics not only defines tumors in a biological manner, but also provides a map for high‐risk regions and intratumor regions that can potentially modulate immune responses, through which it can guide function‐based radiation planning and delivery.

The biology of radiation on the human body is not yet fully understood. Radiation therapy is generally believed to exert its therapeutic effects exclusively locally within the irradiated field to cause direct and indirect cellular DNA damage.^57^ A great deal of radiobiology research was dedicated to DNA damage. Distant effects of radiation were found in the 1950s, and termed the abscopal effect by Mole from the Latin words “ab” and “scopus.”^58^ The abscopal effect refers to the regression of metastatic cancer outside of the irradiated field, which suggests that the immune system is modulated to combat cancer in the whole body as a result of the local therapy.

Radiation therapy was initially considered immunosuppressive, owing to the sensitivity of lymphocytes to radiation and the potential of killing tumor‐infiltrating lymphocytes by radiation therapy.^59^ Radiation is associated with the depletion of circulating lymphocytes^60^ and chronic toxicities.^61^ A study showed that higher radiation doses to the circulating immune cell pool as a surrogate were associated with worse outcomes, such as increased tumor progression and death,^62^ when analyzing radiation to immune cells in patients with stage III NSCLC undergoing conventional definitive radiotherapy (median dose of 60 Gy; 60% of patients underwent intensity‐modulated radiation therapy). The results showed that conventional whole tumor irradiation characterized by large volumes and multiple daily fractions, such as fractionated radiation at a median dose of 60 Gy/30 fraction (2 Gy per fraction), caused global hematological immunosuppression and immune‐related late toxicities.

However, radiation therapy has recently been shown to enhance various components of the immune system, including but not limited to: generating neoantigen from tumor after radiation to prime T cells,^63^ facilitating immunogenic cell death‐induced antigen release and pro‐inflammatory signals,^64^ activation of cytokine cascades to activate innate immune response,^65^ and enhancing T‐cell homing, traffic to, and infiltration into tumors.^66^ An active area of investigation is varying doses and fractionation of radiation therapy in combination with various immunotherapy agents, as well as evaluating the antitumor immune response in various tumor types.^67^, ^68^ Recently, several clinical trials investigating the potential of the combined strategy have been reviewed for both stage III and advanced NSCLC.^69^ It is reasonable to hypothesize that radiation could modulate immune functions in its applications, such as dose/fractionation/site/area of delivery. If the hypothesis is proven, radiomics and genomics studies would be valuable to elucidate the effect of radiation on the human body systematically. On occasion, it is difficult or impractical to deliver the higher dose per fraction ideal for eliciting an antitumor immune response, owing to tumor size or location. Under these circumstances, radiation therapy may be delivered by irradiating a fractional tumor volume. Spatially fractionated radiation therapy is a way to deliver an inhomogeneous radiation dose to a whole or partial tumor.^70^ One way of delivering spatially fractionated radiation therapy is called single fraction three‐dimensional lattice radiation therapy (LRT). A study with a mouse xenograft lung tumor model showed that LRT significantly postponed the growth of an unirradiated distant tumor. The same study also showed that infiltration of CD3^+^ T cells increased significantly in the right‐sided un‐irradiated tumor after 50% tumor volume was irradiated with LRT in the left‐sided leg tumor. Compared with whole tumor volume irradiation, CD3 T‐cell infiltration was also observed in the unirradiated tumor after high‐dose partial irradiation with LRT. This showed that the host immune system could be activated in a different way using partial tumor irradiation.^71^

Preclinical studies demonstrated that the hypoxic tumor cells showed a higher abscopal potential than the normoxic cells of the same tumor type.^72^ An interesting animal study showed that targeting hypoxic tumor regions with a radiation boost improved the control of tumors relative to a controlled boost with the same dose to non‐hypoxic regions of the tumor. This shows that small biological distinct subvolumes within a tumor can determine tumor curability.^73^ Another study showed that hemi‐irradiation of the mouse tumor volume was sufficient in controlling the tumor and eliciting CD8^+^ T cell‐mediated immune response to the irradiated tumor; furthermore, partial radiation also significantly postponed tumor growth in the contralateral non‐treated tumors.^74^ One of the attempts to leverage the immunogenic potential of radiation was stereotactic body radiation therapy targeting hypoxic segment of bulky tumors.^75^ It is worth noting that partial tumor irradiation targeted the more immunogenic‐hypoxic clonogenic cells, sparing the locoregional immune cells at tumor edge as an organ at risk. The time synchronization of irradiation can individually determine optimal timing corresponding to the most reactive phase of the immune antitumor response. Radiation, if used appropriately, could aid local and distant radiation‐induced immune‐mediated antitumor responses. Analysis of non‐irradiated abscopal tumor sites showed the strongest signals of cell death‐regulating signaling molecules interleukin‐6, allograft inflammatory factor, and tumor necrosis factor‐alpha, which had higher expression levels compared with the partially irradiated tumors. This suggests potential cell death‐inducing signals in non‐irradiated out of field abscopal sites.^75^

### 3.2 Radiomics‐guided precision radiation therapy

Ling et al. introduced the concept of “biological target volume (BTV)”, which represents a subvolume of the tumor with specific characteristics on functional or molecular imaging techniques.^76^ Radiomics potentially helps explore the heterogeneity of tumor spatial structure and map BTV. Regions identified by radiomics that either confer radioresistance or elicit immune reactions could be tailored for precision radiation planning, thus better local control or induction of systemic immune reactions can be achieved. We envision the concept of BTV inside the gross tumor could be defined by the aid of radiomics and biological information extracted from images, and then used in radiation planning to guide treatment in a systemic biological manner: various BTVs intratumoral and/or TME will be radiated differently to maximize local debulking effects and enhance immunity. Therefore, this concept is leading to radiomics‐guided radiation therapy.^77^

Wu et al. proposed a robust tumor‐partitioning method using a two‐stage clustering procedure,^27^ identified three spatially distinct and phenotypically consistent subregions in lung tumors, and defined the “high‐risk” subregion, which was associated with the most metabolically active, metabolically heterogeneous, and solid component of the tumor. The volume of the high‐risk intratumoral subregion can be used to predict distant metastasis and OS in patients with NSCLC treated with radiation therapy. High‐risk tumor subregions associated with the aggressive disease can also be targeted with a radiation boost to potentially improve local control and patient survival.

Additionally, by controlling the peritumor environment exposure to the proper amount of radiation, clinicians can potentially manipulate the combination of inducing immune responses by irradiating to the interior part of the tumor with a high dose while facilitating and protecting T‐cell infiltration to the tumor with a lower dose on the edge. A lower optimal dose of radiation delivered to the tumor edge might elicit changes in TME, which would potentially enhance immune effects. The immunomodulation of the TME is largely subject to the dose and number of fractions of radiation therapy. High doses of 12–16 Gy X1 have been shown to hinder T‐cell response, whereas lower doses induce the production of interferon‐β required for dendritic cell activation.^77^

Whether single high doses or fractionated low doses would better complement ICIs is still unclear. However, the optimal application of radiation may incrementally improve target delineation and functional clinical outcome.

### 3.3 Potential to incorporate novel biomarkers in clinical trials

Biomarker‐based trials are ongoing in oncology practice. A marker‐related trial includes the following steps: phase I attempts to validate the marker effectiveness, phase II validates the subpopulation based on certain markers responding to the given therapy, and phase III tries to confirm the clinical benefit from makers based on phase II studies.^78^, ^79^

The Biomarker‐integrated Approaches of Targeted Therapy for Lung Cancer Elimination (BATTLE) trial was the first prospective, biomarker‐based trial in patients with pretreated NSCLC. After profiling 11 prespecified biomarkers, patients were assigned to one of the five biomarker groups based on biopsy findings, and then initially equally randomized into one of the four treatment arms without considering their biomarker status. After this phase, patients were randomly assigned to treatment according to the Bayesian adaptive algorithm. The feature of the Bayesian adaptive algorithm is to randomize patients based on probabilities to treatments based on early outcome data. Patients are randomized based on their biomarker profiles into treatments with the highest likelihood of potential for efficacy in the BATTLE trial.^80^, ^81^ The BATTLE‐1 trial established the feasibility of adaptive design in lung cancer treatment and used biomarkers from pretreatment biopsy in NSCLC patients to guide treatment.^82^

One type of clinical trial is an umbrella study, which incorporates biomarkers and clinical information together. Patients are typically subdivided into subgroups based on biomarkers. One of the examples in lung trials is Adjuvant Lung Cancer Enrichment Marker Identification and Sequencing Trials (ALCHEMIST), which applied the umbrella design to identify and screen patients based on histology and multiple biomarkers, such as EGFR, PD‐L1, and anaplastic lymphoma kinase.^83^ Early‐stage completely resected non‐squamous lung cancer patients who were eligible for the ALCHEMIST trial were assigned to different treatment arms based on their EGFR/anaplastic lymphoma kinase/PD‐L1 status. In each arm, patients were randomized to receive targeted therapy or a placebo.^84^ The ALCHEMIST trial using the umbrella platform has the potential to enhance efficiency and provide a precision medicine‐based approach to add new substudies with novel biomarkers in a defined subset of patients.^85^

### 3.4 Potential of radiomic biomarkers in immuno‐oncology trials

Currently, patients are subject to ICIs based on their PD‐L1 status in immune‐oncology trials; however, the outcome study is only based on imaging of RECIST criteria. The current RECIST criteria were based on simple metrics, such as diameters of the lesion/node in the short or long axis, which do not consider the complete information carried with the imaging, such as the shape and heterogeneity of the lesion/node.

One potential direction of the clinical trial is to identify patients based on a series of radiomic biomarkers and subgroup them into different categories: radiomics signatures will be utilized to define tumor aggressiveness and the level of the immune response.^86^ Tentatively, the potential four categories are as follows based on different TME groups with potential implications for mechanism and therapy have been identified according to B7‐H1 (PD‐L1) expression and the presence of tumor‐infiltrating lymphocytes in tumor biopsies.^87^  The first category is hot tumor/adaptive resistance. This group has low aggressiveness or fewer hypoxic regions, but with a potential strong immune response, as manifested by more immune cell infiltration and a relatively active TME. The second category is intermediate (lazy) tumor/a situation of tolerance. This group has low aggressiveness or fewer hypoxic regions, but with weak immune responses, such as less immune cell infiltration or an immunosuppressive microenvironment. The third category is intermediate (active) tumor/balanced situation. The tumor has high aggressiveness and more hypoxic regions exit, yet with a strong immune response with more immune cell infiltration and an immunoactive microenvironment. The fourth category is cold tumor/immunological ignorant. The tumor has high aggressiveness, but a low immune response and has an immunosuppressive microenvironment. After patients are subgrouped based on radiomic signatures, they can enter biomarker‐driven clinical trials where they would be randomized to the immune point inhibitors/placebo to elucidate their treatment response, which is used to validate the radiomic signatures. Once the validity of the signatures is established, patients can be entered into an umbrella trial and randomized to treatments based on the status of radiomic signatures. Chemotherapy and radiation therapy are frequently used to prime tumors and harness the host's immune system to attack tumor cells.^88^, ^89^ Each category will be randomized to monotherapy with ICI, ICI + chemotherapy, ICI + whole tumor radiation therapy, ICI + partial tumor radiation therapy, and control to optimize current therapeutic methods with each biological distinct group. This is a bridge to radiomics‐driven precision medicine.

Radiomics can provide biomarkers and easily predict important clinical measures, such as therapy outcome, stage, grade, and other main biological pathways for selection of the optimal therapy regimen in lung immuno‐oncology; radiomics‐guided radiotherapy will improve the ablation effect and immunomodulatory effects of radiation therapy.

## 4 PERSPECTIVES

Radiomics has the potential to elucidate tumor biology and prognosis in a non‐invasive, comprehensive, and efficient manner. It also has the potential to guide the selection of drug treatment and clinical planning of radiation therapy. The application of radiomics in lung immuno‐oncology will need to be validated in large‐scale prospective studies. Integration of an expert panel of clinical practice, biology, data science, and artificial intelligence would be instrumental in the future development of lung immune oncology.

## ETHICS STATEMENT

This study is not involved in any ethical issues.

## CONFLICT OF INTEREST STATEMENT

The authors declare that they have read the article and there are no competing interests.
